# Surface defect detection method for electronic panels based on double branching and decoupling head structure

**DOI:** 10.1371/journal.pone.0279035

**Published:** 2023-02-24

**Authors:** Le Wang, Xixia Huang, Zhangjing Zheng, Hui Ruan

**Affiliations:** Institute of Logistics Science and Engineering, Shanghai Maritime University, Shanghai, People’s Republic of China; Shandong Normal University, CHINA

## Abstract

During the production of electronic panels, surface defects will inevitably appear. How to quickly and accurately detect these defects is very important to improve product quality. However, some problems such as high cost and low accuracy are still prominent when existing manual detection and traditional techniques are used to solve such problems. Therefore, more and more computer vision techniques are proposed to solve such problems, but the current application of deep learning-based object detection networks for surface defect detection of electronic panels is in a gap. The analysis found that there are two main reasons for this phenomenon. On the one hand, the surface defects of electronic panels have their unique characteristics such as multi-scale and irregular shape, and the current object detection networks cannot effectively solve these problems. On the other hand, the regression and classification tasks coupled in the current computational mechanism of each network are commonly found to cause the problem of conflict between them, which makes it more difficult to adapt these network models to the detection tasks in this scenario. Based on this, we design a supervised object detection network for electronic panel surface defect detection scenario for the first time. The computational mechanism of this network includes a prediction box generation strategy based on the double branch structure and a detection head design strategy that decouples the regression task from the classification task. In addition, we validated the designed network and the proposed method on our own collected dataset of surface defects in electronic panels. The final results of the comparative and ablation experiments show that our proposed method achieves an average accuracy of 78.897% for 64 surface defect categories, proving that its application to electronic panel surface defect detection scenarios can achieve better results.

## 1. Introduction

During the production of electronic panels, defects such as scratches, cracks, dents and bumps, foreign colors, bright spots and black spots will inevitably appear due to the precision error of production equipment and oper1ator error, etc. Detecting these defects in a timely manner is very important to improve the yield of subsequent products and enhance customer experience. The detection of surface defects in electronic panels by machine vision methods is a very specific and highly applied object detection problem. The detection process involves surface defect image data processing, feature extraction, and the design of detection head. All these modules together form a complete object detection network. In recent years, deep learning-based target technology has developed rapidly. In terms of network structure [1–4], proposed two stage series of object detection networks and [5–9] proposed one stage series of object detection networks. The two stage series object detection network first feeds the input image into the first stage RPN (region proposal network) network to generate the region of interest, and then the second stage is used to predict the target more finely. The RPN network is mainly used to filter out possible target regions as well as to predict partial targets, and then output the final prediction by combining it with the prediction results of the second stage network. Higher accuracy can be obtained in this way, but at the expense of detection speed to a large extent. The one stage series object detection network learns all the generated anchors directly, which greatly speeds up the detection speed. But one stage series object detection network learns the anchor features that are unfavorable to the network, resulting in a less accurate than the two stage series network of the same period. The Current mainstream object detection networks have achieved good results on public datasets such as MS COCO [10] and PASCAL VOC [11]. For example, Dai et al. [12] proposed resnext-101-DCN (Deformable Convolution) backbone with its designed dynamic head, which achieved an AP of 54.0 in MS COCO [10]. Zhang et al. [13] proposed a DINO(DETR with Improved DeNoising Anchor Boxes for End-to-End Object Detection) network by using a comparison method for denoising training, mixed query selection for anchor initialization and a look forward twice scheme for regression box prediction. The method also achieved 48.3 AP at the 12th epoch and 51.0 AP at the 36th epoch on MS COCO [10] using ResNet-50 as the backbone.

**Fig 1:**
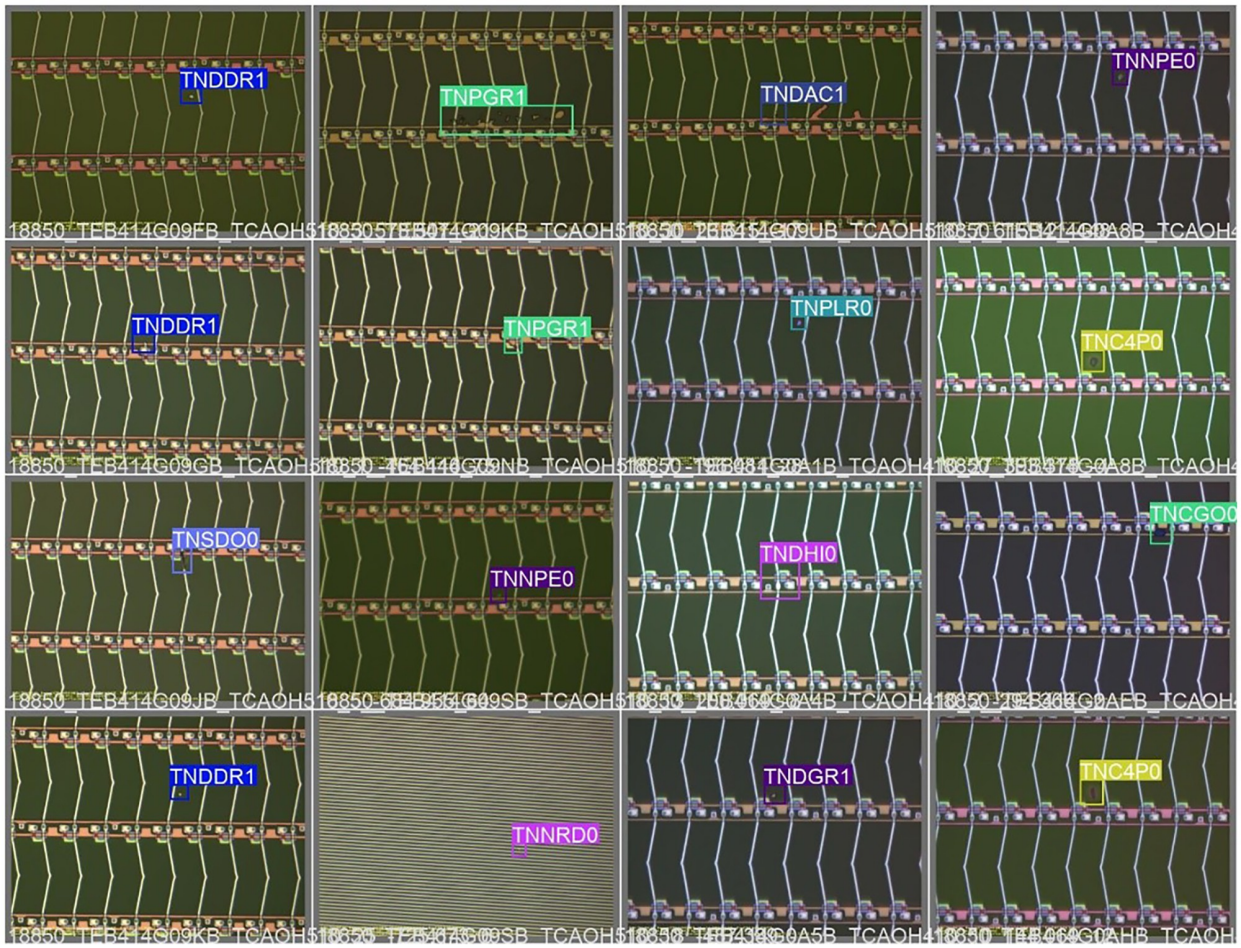
Common electronic panel surface defects.

These object detection networks have achieved good results in their respective experiments, but they are all designed specifically for public datasets. When these methods are directly applied to datasets from other domains, the problem of insufficient generalization performance of the models and poor detection results often occurs. This is because the distribution of data features in different scenes is inconsistent and the weights of the networks obtained from training vary too much. In recent times, numerous scholars have proposed improvements to these networks for use in their own datasets. Xie et al. [14] designed a two-branch convolutional neural network for cloud detection in weather forecast analysis. Zhu et al. [15] improved the detection head of YOLOV5 with transformer and applied it to UAV detection of street targets. Jing et al. [16] combined MobileNet [17] and U-Net [18] networks and designed median frequency balancing loss function for detecting fabric defects. However, no object network has been proposed for the detection of surface defects in electronic panels. The main reason is that the defect targets of electronic panels are quite different from those of other fields. The surface defects of common electronic panels are shown in Fig 1, which exhibit three main characteristics:

1. Electronic panel surface defects are only available in anomalous cases, making the overall sample size small as well as the category distribution unbalanced. These two cases caused overfitting and biased learning of common defect features while ignoring information of rare defect features during the training process. The training of the object detection network becomes very inefficient as a result.
2. Small target type is more, such as black spots, bright spots, etc. The small size of small targets causes their available features to be limited. Also, since their semantic information appears in shallower feature maps, their detailed information may disappear completely as the network deepens, making them extremely ineffective in the regression task and often miss detection.
3. The multi-scale problem of defects is obvious, specifically irregular size, extreme aspect ratio and variable shape, such as scratches, cracks and other defects. The current object detection network is difficult to effectively extract the features of these types of defects, which can easily lead to false and missed detections.

Meanwhile, in the current computational mechanism of object detection networks, anchor-based or anchor-free single prediction box generation strategies are widely used, as well as detection strategies that are deeply coupled between regression tasks and classification tasks. The deficiencies of the network model are further amplified by these computational mechanisms in the face of the unique characteristics of surface defects in electronic panels, mainly in two aspects:

1. The generation strategy of the prediction box and the feature selection mechanism are not optimal for the detection of multi-scale targets by the single-anchor-based mechanism and the anchor-free mechanism. When this strategy is used for the detection of small and multi-scale targets in electronic panel surface defects, the phenomenon of missed and false detection is very likely to occur.
2. The two tasks of regression and classification conflict with each other and affect the effectiveness of their respective tasks. In particular, the degree of this conflict is further deepened by the subtle differences between the surface defects of the electronic panels and the background, which are manifested in all defect type targets.

In the combination of the above factors, the current object detection methods are difficult to achieve better detection results in the detection of surface defects in electronic panels, and thus there has been a lack of research results with better results. To address these problems, we designed a supervised object detection network for surface defects in electronic panels. In the design process, the use of anchor-based and anchor-free dual branching structures is proposed for prediction box generation and feature selection. The decoupled detection head is designed at the detection side for object classification and regression. The object detection network we designed can be applied to small-target, multi-scale electronic panel surface defect scenarios.

In summary, our contributions are mainly in the following aspects:

1. Developed a dataset of electronic panel surface defects. The images in our dataset come from the real environment of the factory, with a total of 6525 images and 64 defect categories.
2. In order to solve the multi-scale and small target problem for surface defect targets of electronic panels, the anchor-based and anchor-free dual branch structure is used for prediction box generation and feature selection in the supervised object detection network.
3. Aiming at the coupling problem of regression and classification tasks in the object detection network, the structure of decoupling head is designed, and the attention mechanism is added to the detection head, which effectively reduces the mutual conflict between regression and classification tasks in object detection network.

In this paper, the first part explains the characteristics of surface defects in electronic panels and some challenges faced by existing object detection methods for surface defect detection in electronic panels, as well as our contribution. In the second part, some surface defect detection methods, as well as anchor-based and anchor-free computer mechanisms, are presented. The third section focuses on our method, including the core network structure and loss function. In the fourth section, a comparison with six methods and ablation experiments are performed to demonstrate the effectiveness of our method. In Section 5, we conclude the paper.

## 2. Related work

In this section, relevant methods for defect detection will be presented, including supervised and unsupervised defect detection methods, and secondly, anchor-based and anchor-free object detection methods will be presented.

### 2.1 Surface defect detection method

The current surface defect detection methods can be divided into supervised surface defect detection methods, unsupervised surface defect detection methods and semi-supervised surface defect detection methods according to data types. Most supervised surface defect detection methods, such as [19–23], are improved on the object detection network developed for public datasets. Among them, it is worth mentioning the end-to-end method proposed by He et al. [19] for steel plate defect detection, which increases the richness of the extracted features by using a feature fusion strategy, but does not address the problems such as small samples and small targets in the field of surface defect detection. Wang et al. [20] proposed a joint detection CNN (Convolutional Neural Networks) architecture consisting of a global framework and a sub-frame framework to detect cloth defects. Compared with traditional algorithms, its detection performance has been greatly improved, but it is still not optimal. Qiu et al. [21] adopted a fully convolutional (FCN) method for pixel-level prediction of defect regions, while using depthwise, pointwise convolutional layers, strided depthwise convolutional layers and up-sampled depthwise convolutional layers to replace the standard convolutional layers layer, pooling layer, and deconvolution layer. This method provides a significant improvement in detection accuracy and detection performance, but since the proposed algorithm is based on local information, its detection capability on structural defects is weaker than that on texture defects.

In order to solve the problem of insufficient defect samples and difficulty in defect feature extraction in surface defect detection [22, 24–27], proposed an unsupervised surface defect detection method. The core idea of these methods is to detect the defect target as abnormal target. Among them, Mei et al. [24] proposed a training method to detect and localize defects using only defect-free samples for fabric defect detection, which is mainly achieved by reconstructing defect-free images with convolutional noise reduction self-encoder networks. This method effectively solves the problem of small samples in a specific scene, but the generalization performance of the model is poor. When the same class of defects exhibits different shapes, the defect class is sometimes not detected accurately. Zhao et al. [22] proposed an unsupervised method for fabric defect detection, constructing a reconstruction model through an autoencoder and a GAN (Generative Adversarial Networks) network, and then feeding the defective image into the model to obtain a defect-free image. Then, the defect location can be obtained by comparing the restored defect-free image with the original image pixel by pixel. This method only needs normal samples for training, and has a good application prospect in project landing. However, due to the inevitable introduction of noise during network reconstruction, the detection accuracy and detection speed still need to be improved.

Although the unsupervised defect detection method solves the problem of insufficient defect samples, it also sacrifices the detection accuracy and detection rate to a certain extent, and the generalization ability of the model is insufficient. Based on this [25, 28, 29], proposed a semi-supervised defect detection method. For example, Di et al. [25] proposed a semi-supervised learning method combining convolutional autoencoder (CAE) and semi-supervised generative adversal network to classify defect samples. Compared with the traditional method, the CAE-SGAN method can make full use of the sample images (labeled and unlabeled images) of the steel surface, which improves the defect classification accuracy under limited training samples. However, the performance of this method is not optimal, and there are still many deficiencies in the location of defects. Gao et al. [28] proposed a semi-supervised learning method using convolutional neural network (CNN) improved by pseudo-label to identify steel surface defects. The method has achieved good detection results on the dataset of a steel company, but the generalization performance of the model is poor. Although semi-supervised learning solves some problems and has some applied research, there is still a lot of room for improvement in the detection of surface defects of electronic panels.

### 2.2 Anchor-based and anchor-free mechanisms

The current computational mechanism of object detection network can be divided into anchor-based mechanism and anchor-free mechanism according to whether anchor box is used in the generation process of prediction box. Anchor box is a commonly used prior strategy for generating prediction boxes in object detection networks. It has been widely used after it was first proposed by Ren et al. [3], and then the classical networks [4, 6–9, 30, 31] both use the anchor-based mechanism. The current anchor-based mechanism has mature technology and high detection accuracy. The anchor-based object detection algorithm needs to determine the scale and aspect ratio of the anchor box, the number of generated anchor boxes, the intersection ratio threshold, and other hyperparameters based on the distribution of the object’s size and aspect ratio in the training data. The constraints of numerous hyperparameters enable the object detection network to learn more information about the objects. Generally speaking, the more the number of selected anchor boxes, the richer the information learned by the network and the better the detection effect. However, the selection of these hyperparameters will directly affect the final accuracy, so this method not only relies on prior knowledge but also lacks generalization ability. In addition, the anchor-based object detection algorithm also produces a lot of computational redundancy. Meanwhile, because of the imbalance of positive and negative samples in the training sample, it will cause low detection accuracy for anomalous objects and multi-scale targets. In addition, due to the need to screen a large number of generated anchor boxes at the detection end, the complexity of the detection head is undoubtedly increased. When making a large number of predictions on some edge AI systems, for example from the NPU to the CPU, it may cause an overall latency bottleneck.

**Fig 2:**
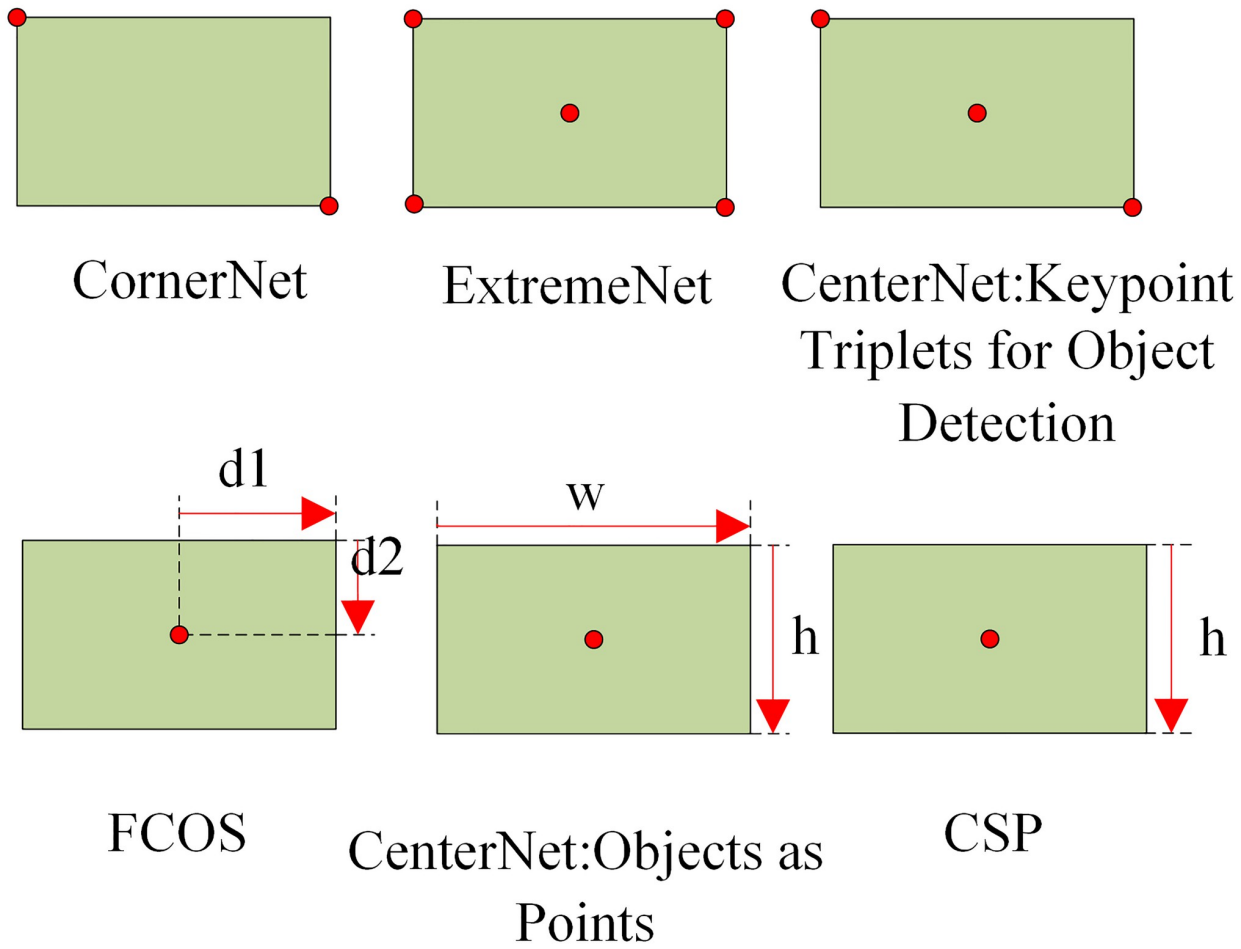
Predictive approach of anchor-free mechanism.

Based on this, the anchor-free mechanism is proposed as a counterpart to the anchor-based mechanism. This mechanism does not use a priori strategy in the generation of prediction box, and directly uses feature information to generate prediction box. The first anchor-free network was proposed by Redmon et al. [5], followed by the classical structure of anchor-free proposed by [32–36], which has been heavily cited, and these structures are embedded in various backbone networks. Achor-free mechanism predicts the center of an target by incorporating the center prediction into the target of the category prediction. For the prediction of four sides, it is more consistent to predict the distance from the pixel to the four sides of the ground truth box, although some tricks are used to limit the range of regress. CornerNet proposed by Law et al. [33] and ExtremeNet proposed by Zhou et al. [34] and CenterNet(Keypoint Triplets for Object Detection) proposed by Duan et al. [36] belong to the joint prediction methods based on multiple keypoints. The joint prediction method based on multiple key points generates the bounding box in three ways: upper left corner point plus lower right corner point, 4 extreme points plus center point, and upper left corner point combined with lower right corner point with center point. FCOS(fully convulsion one-stage object detector) proposed by Tian et al. [35], CenterNet(Objects as Points) proposed by Zhou et al. [37], and CSP(Center and Scale Prediction) proposed by Liu et al. [38] are single-centroid-based prediction methods. These methods generate the bounding box by center point to box distance, center point plus width and height, and center point plus height, respectively. The prediction methods of various anchor-free mechanisms are shown in Fig 2. Anchor-free exhibits strong generalization ability, more concise framework, high accuracy of anomaly-scale object detection, suitable for small object detection, and can greatly reduce the number of parameters in the network. However, the anchor-free approach is not suitable for generic object detection and has lower accuracy than the anchor-based algorithm.

## 3. Method

In this section, a supervised object detection network for electronic panel surface defect detection is proposed, aiming to improve the detection of small target and multi-scale target defects for electronic panel surface defect detection, including the use of anchor-based and anchor-free double branch structure in the prediction box generation and feature selection process, and the decoupled detection head used at the detection end will be described in detail.

### 3.1 Network architecture

**Fig 3:**
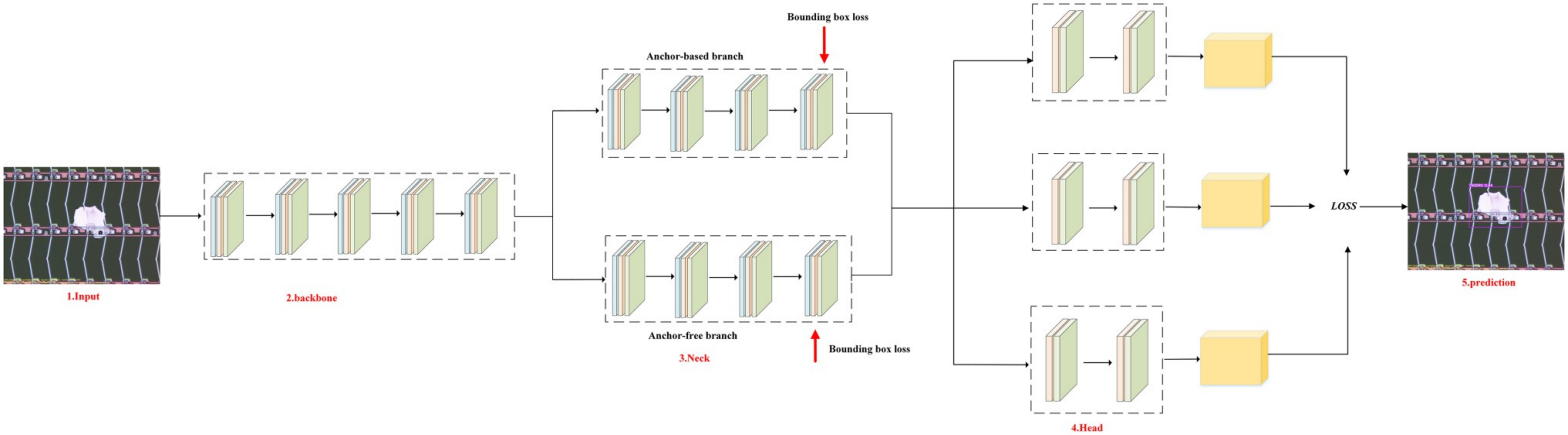
Overall network structure. Surface defect detector for electronic panels based on double branching and decoupling head structure. This includes backbone, neck which uses double branch for prediction box generation, and decoupled detection heads that incorporate attention mechanisms, including three large detection heads, each of which is decoupled into a classification detection head and a regression detection head. The details will be described in chapter 3.

As shown in Fig 3, considering that a large number of electronic panel products often need to be detected quickly in a short period of time in the later production process, the detection speed of the network is required to be high. Our network structure uses numerous components from the one-stage framework, on the basis of which we redesign the generation strategy of the prediction box and the detection head of the decoupling structure. These structures are designed mainly for the small and multi-scale targets of electronic panel defects, the coupling of regression and classification tasks and the subsequent engineering practical deployment in real time. In general, our network structure consists of backbone, neck, and head. Backbone uses focus and csp structures, mainly to keep the sampled feature information from being lost, as well as to reduce the size of the model while ensuring inference speed and accuracy. Neck adopts the fpn and pan structures in order to better utilize the feature information extracted by backbone. In particular, we embed the double branch module in neck, which is designed for the characteristics of small and multi-scale targets of electronic panel surface defects. This structure enables the network’s prediction box generation strategy to have strong generalization performance in predicting small targets, regular size targets, and multi-scale targets for electronic panel surface defects, avoiding false and missed detections of small and extreme size targets. For the detection heads of the defect detection network, a specially designed decoupled head structure is used. Specifically, there are three detection heads, each of which is decoupled into a classification subhead and a regression subhead. This decoupled structure improves the detection effect in all types of instance targets compared to the coupled structure.

### 3.2 Double branch structure

Object detection, as a multi-task problem, requires accurate prediction of both class and location information of the target. In the inference stage of the object detection network, it is necessary to compare the prediction box with the actual location of the target by generating a prediction box, so as to continuously optimize the network parameters and make the prediction box closer to the ground-truth location of the target. Anchor-based, as a relatively mature prediction box generation strategy in the current object detection network, is widely used in common networks such as Faster-RCNN [3], YOLOV2 [6], YOLOV3 [7], YOLOV4 [8], SSD [9]. Anchor generates a series of rectangular boxes as prediction boxes through prior knowledge or clustering methods, and then slides in the image window, and then retains the part of the prediction box that is closest to the actual position of the target through the NMS (non maximum suppression) strategy. The prediction box generated by this strategy can learn a wealth of information and achieve better detection results through the constraints of many parameters when faced with conventional scale targets. However, in the face of multi-scale targets, it is very easy to cause false detection and missed detection. Especially in the surface defects of electronic panels, the prediction box generated by anchor-based can only cover the targets of conventional scales, but cannot form effective correspondence with small targets and extremely large targets. At the same time, the features selected by the anchor matching mechanism are not optimal, and this disadvantage is especially obvious in multi-scale targets, because the learning effect of multi-scale targets is determined by the semantic information most relevant to its category features. Therefore, the anchor-based strategy is suitable for conventional scale targets, but it cannot be applied to the detection scenes of small targets, extreme targets and multi-scale targets with electronic panel surface defects, which will cause frequent leakage and false detection phenomenon to occur. This will be indicated in the comparative experiments. However, another anchor-free method does not rely on pre-defined anchor boxes and directly generates prediction boxes. This method relies on the key point or center point in a pixel location on the feature map to directly predict the regression target (ground-truth). Although the accuracy of the anchor-free method is not as good as that of the anchor-based one, and it is not suitable for general-purpose object detection. However, its simpler framework and high accuracy of anomalous scale object detection can be applied to multi-scale object detection and small object detection, and it can just play its advantages in the electronic panel surface defect detection scenario.

**Fig 4:**
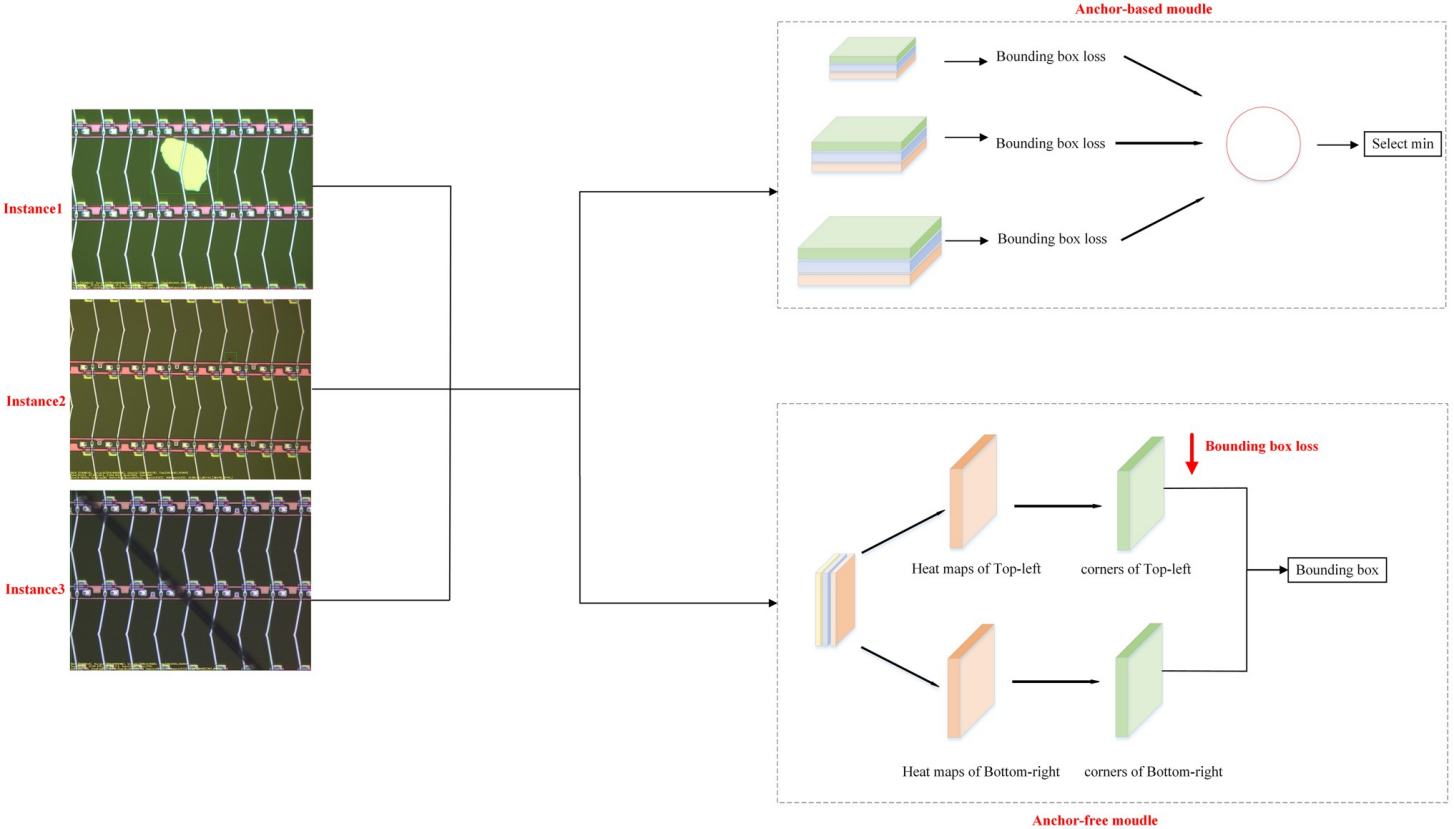
Double branch structure.

For the respective advantages and disadvantages of anchor-based and anchor-free, as well as the characteristics of electronic panel surface defects, we designed double branch, whose structure is shown in Fig 4. The double branch module is composed of two branches, anchor-based branch and anchor-free branch. The anchor-based branch is used to solve the detection problem of conventional scale targets within a certain range. The anchor-free branch is used to solve the detection problem of small targets, extreme size targets, and multi-scale targets. This combination can effectively enhance the redundancy capability of the network in the face of multi-scale targets and improve the detection accuracy of the network, making the designed network better suited for the electronic panel surface defect detection scenario. Specifically, in the prediction box generation strategy, we propose anchor-based and anchor-free double branch structure. In the anchor-based branch, we have further improved the feature matching strategy. Instead of using the NMS strategy in many object detection networks for selection, the regression loss between the generated prediction box and the target’s true location box is directly calculated. The feature map corresponding to the prediction box with the smallest regression loss is used to guide the selection of the most suitable semantic information for that target level, so as to achieve the optimal matching of features and thus improve the effectiveness of object detection.

For the anchor-free branch, it mainly completes small and extreme scale targets that cannot be detected by the anchor-based branch. In this branch, in order to achieve a balance between reducing parameters and maintaining accuracy, we refer to CornerNet [34], which currently works best, to generate multiple key points of the prediction box directly at the upper left corner point corresponding to the target feature map and the lower right corner point. Here, regression loss between the prediction box and the ground-truth target position box is selected as regression loss which is same in the anchor-based branch. The advantage of this method is to facilitate the ground-truthization of unified fusion between the two branches. In this section, our innovations are mainly the following two points: (1) The feature-guided matching mechanism in the anchor-based branch is redefined to achieve the most reasonable selection of features for the target. (2) The dual branch structure of anchor-based and anchor-free is designed to complement the advantages of both, so that the designed network can be applicable to the detection of various scale targets in the detection of surface defects in electronic panels. As shown in Fig 3, our network is able to achieve the best match in detecting electronic panel surface defect targets at three scales of instance 1, instance 2, and instance 3.

### 3.3 Decoupled head with attention mechanism

**Fig 5:**
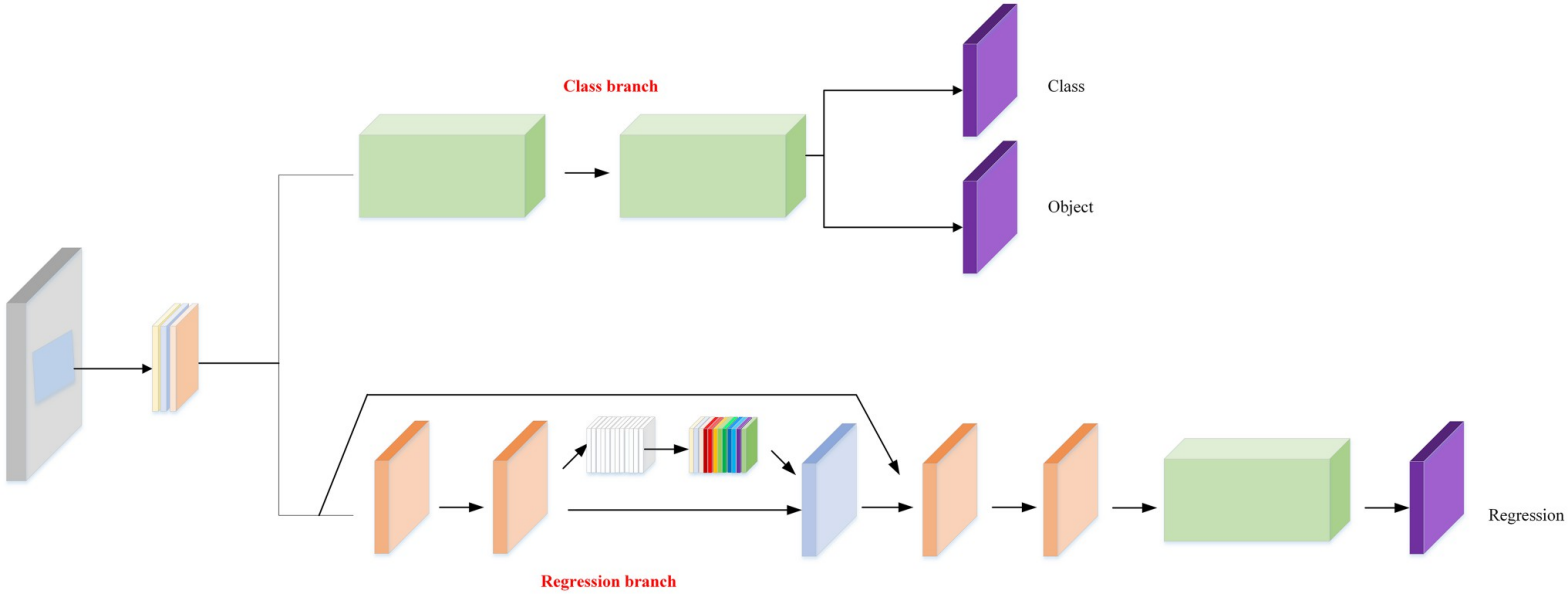
Decoupled head structure with attentional mechanism.

In object detection networks, the conflict between classification and regression tasks is an ongoing problem. The reason for this is analyzed because the classification and regression tasks share the weights of the whole neural network. However, the information of the weights required by each of the two tasks of classification and regression is inconsistent, and it is difficult to find suitable parameters to balance the learning preferences between the two tasks in the optimization process of the network. This is still the case when most of the currently adopted improved two-stage and one-stage object detection methods are used for the detection of surface defects in electronic panels. To address these problems, Song et al. [39] and Guo et al. [40] proposed the idea of feature decoupling to decouple the features needed for the classification task and the regression task for extraction, but some information is lost in the process of decoupling. In addition, there are attempts to decouple in the detection head in one-stage networks and two-stage networks, but the joint optimization of the two types of tasks is still being performed in the final stage using a uniform loss function, which is not a ground-truth decoupling. Based on this, we redesigned the new decoupling head by combining the respective characteristics of classification task and regression task, and the specific structure is shown in Fig 5.

Specifically, we still use the detection head structure of YOLOV4 and YOLOV5 in the overall structure, and use three scales of detection heads to detect defect targets of different scales. However, in the specific design of each detection head, we decoupled each large detection head into a classification task head and a regression task head. Classification loss is used to optimize the classification head and regression loss is used to optimize the classification head to achieve a decoupling effect between the two types of tasks. In addition, based on the selection of different feature information for classification tasks and regression tasks, we redesigned the specific structures of the classification subhead and regression subhead to improve the decoupling effect. First of all, in most classification networks, the last layer of network structure adopts the fully connected layer. Because the convolutional layer extracts local features, the full connection is to reassemble the previous local features into a complete graph through the weight matrix, and the label category with the highest output probability is used as the predicted category. Therefore, the fully connected layer is a classifier, and its effectiveness in classification tasks is also better than that of convolutional layers. At the same time, in the experiments of Wu et al. [41], it is also proved that the classification effect of the fully connected layer is better than that of the convolutional layer. Therefore, we replace the original convolutional layer with a two-layer fully connected layer structure as the structure of the classification subhead in our decoupled head. For the regression task, it only focuses on the feature information related to the target position. Whether the feature information related to the position can be accurately transmitted into the detection head determines the prediction effect of the regression task. However, the local feature obtained by the convolution layer cannot completely correspond to its position information. In response to this, we embed the coordinate attention moudle before the convolutional layer. The central focus of the attention mechanism is to get the network to pay attention to what it needs to pay more attention to. When we use a neural network to process images, we would prefer that the neural network pay attention to what should be paid attention to, rather than to everything. Therefore, it becomes extremely important to get the convolutional neural network to pay attention to important objects adaptively, and the attention mechanism is one way to achieve adaptive attention of the network. The coordinate attention moudle can help the regression subhead accurately capture position-related feature information by embedding position information into the channel attention. In addition, for the confidence branch, since it is related to the classification task, the confidence branch and the classification branch share the weights of the fully connected layers. Through the above redesign of the two subheads, the decoupling of the regression task and the classification task is ground-truthized.

### 3.4 Loss function

In the electronic panel surface defect detection network designed by us, the prediction box and ground-truth regression loss, defect classification loss, and confidence loss function are used to constrain the network, which will be introduced in detail in this section.

#### 3.4.1 Localization loss

In the sub-detection head of regression task, regression loss is used to constrain the error between the prediction box and the ground-truth box, so that the prediction box is constantly close to the ground-truth box. Meanwhile, localization loss here is also used for fusion in the anchor-based and anchor-free double branch structure. Due to the multi-scale and small dataset of electronic panel surface defects, we combined the α-IOU bounding box loss function proposed by He et al. [42], and designed regression losses are shown as follows:

$$L_{1}=1-IOU^{\alpha }+\frac{\rho^{2\alpha }\left(b,b^{gt}\right)}{c^{2\alpha }}+{\left(\beta \upsilon \right)}^{\alpha }$$ (1)

Where

$$\nu =\frac{4}{\pi^{2}}{\left(tanh\frac{w^{gt}}{h^{gt}}-tanh\frac{w}{h}\right)}^{2}$$ (2)

$$\beta =\frac{v}{\left(1-IOU\right)+v}$$ (3)

$$IOU=\frac{\left|b\cap b^{gt}\right|}{\left|b\cup b^{gt}\right|}$$ (4)

Where them, *b* and *b*^*gt*^ represent the predicted box and the ground-truth box respectively, *ρ*^2^(*b*, *b*^*gt*^) represents the euclidean distance between the predicted box and the ground-truth box, and *c*^2^ represents the square of the distance from the upper left corner to the lower right corner or the lower left corner to the upper right corner between the predicted box and the ground-truth box. In addition, *w* and *h* denote the width and height of the predicted box, respectively, and *w*^*gt*^ and *h*^*gt*^ denote the width and height of the ground-truth box, respectively. And *α* is a hyperparameter. According to the experimental results in the literature [14], we take the optimal value of *α*, 3, as the hyperparameter of *L*_1_ in our experiments.

#### 3.4.2 Classification loss and confidence loss

In the object detection task, the classification loss function is used to guide the learning of the classification task, which can be expressed as:

$$L_{1}=-\frac{1}{n}\sum \left(y_{n}\times lnx_{n}+\left(1-y_{n}\right)\times \text{ln}\left(1-x_{n}\right)\right)$$ (5)

Where them, n represents the total number of categories, *x*_*n*_ is the predicted value of the current category, and *y*_*n*_ is the label 0 or 1, since the dataset selected in this experiment contains 64 types of defect samples, so n is 64, and sigmoid operation needs to be performed before the incoming value *x*_*n*_, the sigmoid formula is as follows:

$$S\left(x\right)=\frac{1}{1+e^{-x}}$$ (6)

### 3.5 Other metrics

To further enhance the detection effect of the designed network on surface defects of electronic panels, we also use a series of tricks that are effective for improving the network. It includes super-resolution preprocessing operations for poor image quality, data enhancement operations for the combination of mixup and mosaic with less training data. In addition, we further expand the dataset by croping defect targets and randomly copying them to non-defective samples, an oversampling operation is performed on defect categories with a sample size of less than 100 to solve the class imbalance problem. These effective tricks all improve the detection effect of our network on the surface defects of electronic panels to a certain extent.

## 4. Experiments

In this section, we use the labeled electronic panel surface defect dataset to compare our method with the most advanced object detection algorithms. Including two-stage and one-stage object detection algorithms, anchor-based and anchor-free object detection algorithms. In addition, effective ablation experiments have been carried out, the specific contents of which will be introduced in detail in this part.

### 4.1 Datasets

Our dataset is collected at the industrial site, and a total of 6155 electronic panel pictures are were captured by vertical angle, and labeled according to the PASCAL VOC data set format using the labelImg object detection data set labeling software. In total, among the 6155 images, there are 370 defect-free images and 5785 defective images, with a total of 64 defect categories. The training set, validation set, and test set are divided according to the ratio of 8:1:1. And the training set, validation set, and test set all contain defective images of each of the 64 defective classes.

### 4.2 Comparisons with the state-of-the-art

**Fig 6:**
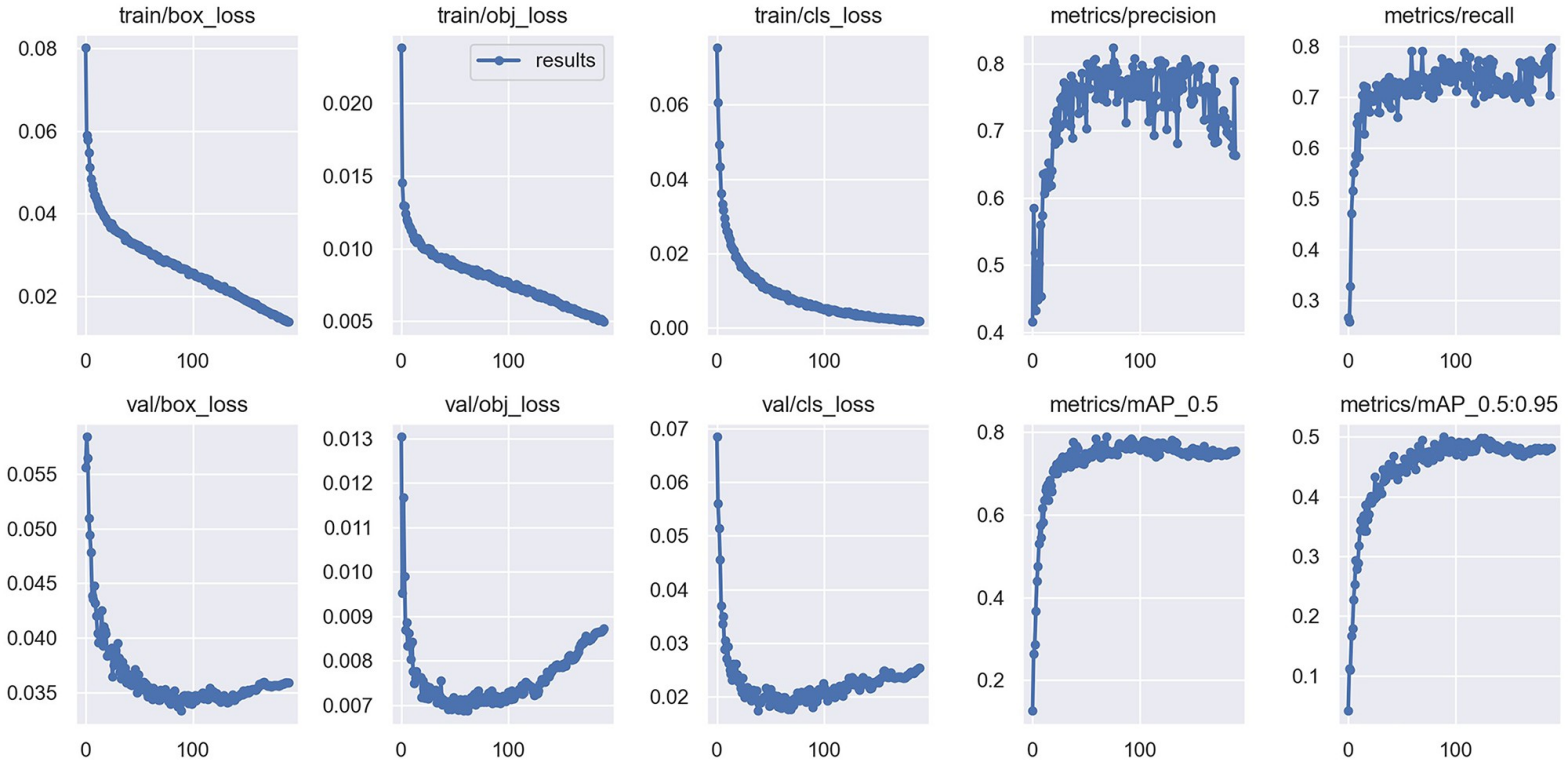
Training results of our designed network.

**Fig 7:**
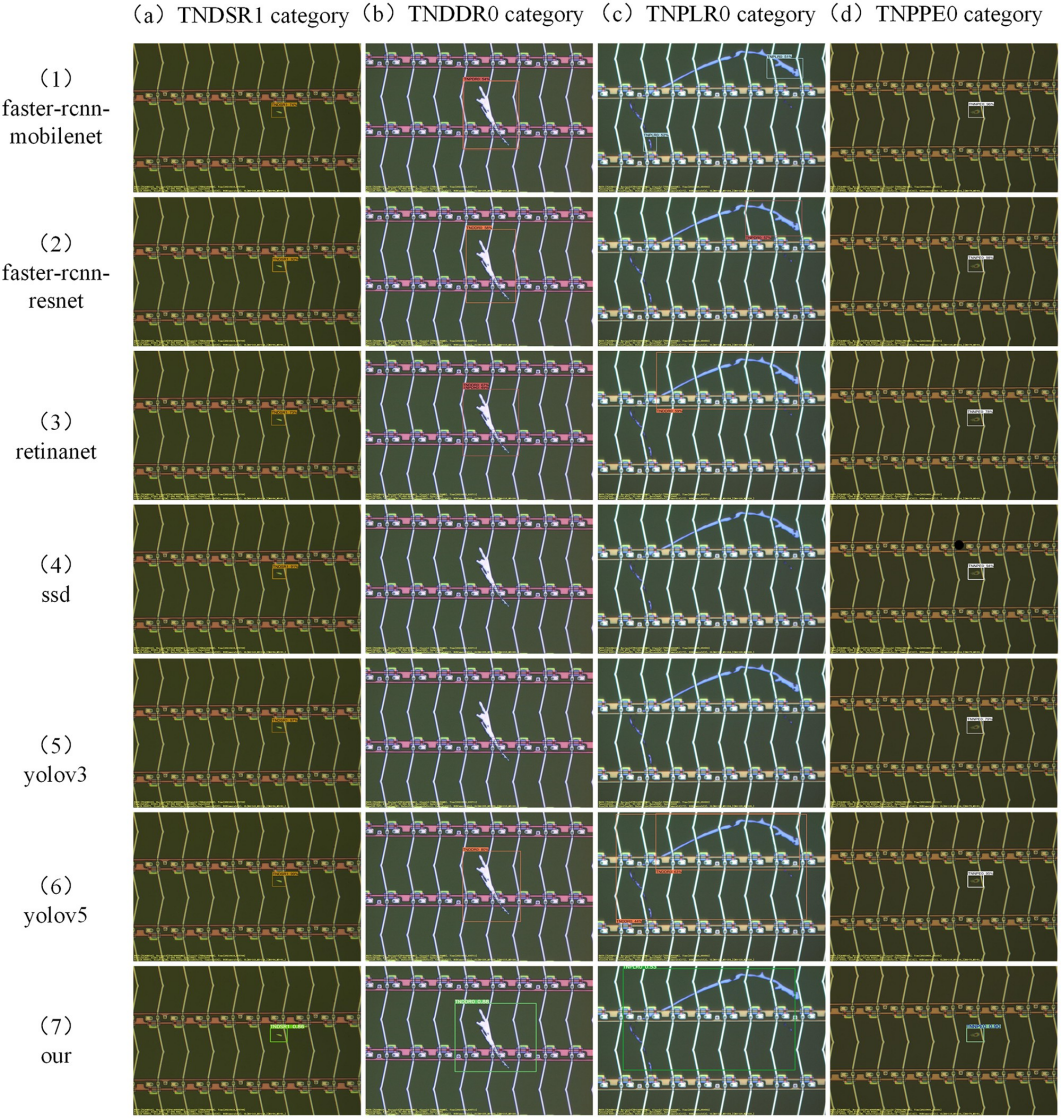
Four images in the TNSDR0 defect category are randomly selected from the test set and tested on our network and several state-of-the-art networks for comparison experiments. From top to bottom, they are Faster-rcnn (mobilenet), Faster-rcnn (resnet), retinanet, SSD, YOLOV3, YOLOV5, and our method.

In order to prove the effectiveness of our proposed network, the designed network is compared with the current state-of-the-art object detection algorithms on the same dataset, including Faster-RCNN, Retinanet, SSD, YOLOV3, YOLOV5 and YOLOX. The experimental results show that our network performs better than several other networks on both the map metric and the recall metric. We conduct all our experiments on the Pytorch 1.9.1 deep learning network framework. The network is trained on a PC with Nvidia GeForce RTX 2060 GPU. We trained the same number of times for each group of experiments, all for 200 epochs, the batch size was 4, and the size of the input image was cropped to 640*640. We used the most widely used map metrics and recall metrics in the field of object detection to measure the effectiveness of defect detection. The training results of our designed network are shown in Fig 6, and the comparison results with the metrics of each network are shown in Table 1. In addition, four images were randomly selected from the test set belonging to the TNSDR0 category, containing small target, pair of scale target defects, and tested with the weights obtained from the training of each network. The detection results are shown in Fig 7.

**Table 1:** The comparison of the performance in our datasets with the state-of-the-art.

method	map_0.5	map_0.5:0.95	recall
Faster-rcnn (resnet50)	0.3828	0.2167	0.3752
Faster-rcnn (mobile-net)	0.3745	0.2116	0.3383
retinanet	0.448	0.263	0.539
SSD	0.3095	0.1843	0.3143
YOLOv3	0.3684	0.22881	0.35226
YOLOV5-S	0.64751	0.39064	0.68898
YOLOX-S	0.6387	0.391107	0.67236
Ours	0.78897	0.49976	0.79689

From the experimental results in Table 1, it can be seen that the algorithm proposed in this paper outperforms the current state-of-the-art object detection algorithms in both map metrics and recall metrics. Our algorithm improves 47.947 percentage points over SSD in the map_0.5 metric and also 10.618 percentage points over the YOLOV5-S network, and our method performs optimally on the training set. Fig 7 shows the detection of different four images in the same defect category with the best weights obtained from the training of each network, containing small target defects and multi-scale target defects, respectively. In the test results, for the small targets in (b), only Faster-RCNN(resnet), Retinanet, YOLOV5 and our method predict correctly, Faster-RCNN(Mobile-net) has a missed detection, and SSD and YOLOV3 have a false detection. For the multiscale targets in (b), (c), and (d), Faster-rcnn (mobilenet), Faster-rcnn (resnet), retinanet, SSD, YOLOV3, and YOLOV5 all showed different degrees of false detection, missed detection, repeated detection, and inaccurate localization, while our method showed different degrees of false detection, missed detection, repeated detection, and inaccurate localization in these three defects, both defect categories and defect locations were accurately predicted, and the confidence levels were 93%, 92%, 92%, and 88% in the four defects, respectively.

The reasons for this are mainly twofold. First, the surface defects of electronic panels present small target and multi-scale characteristics, and each network in the comparison experiments is an anchor-based or anchor-free object detection network. The single-anchor-based method has difficulty in forming effective mappings of the set anchor sizes to extremely small and extremely large scale targets due to insufficient a priori knowledge. This can result in difficulties in accurately learning the feature information of defects at these scales, and thus the phenomenon of missed detection. Meanwhile, the anchor-free mechanism is less effective in the detection of regular targets due to fewer constraint parameters. The dual-branch structure we have designed complements the advantages of the anchor-based and anchor-free mechanisms. The anchor-free branch can better solve the detection of extreme small-scale targets and large-scale targets compared to the anchor-based mechanism, reducing the probability of false and missed detection of small and large targets. The improved anchor-based branch further optimizes the screening of anchor by boudingbox loss, which also improves the detection accuracy of regular scale targets and reduces the probability of false detection of common size targets to a certain extent. Second, each network in the comparison experiments has a certain degree of coupling at the detection head end, which causes conflicts between regression and classification tasks and affects the overall performance of the object detection network. The decoupled head we designed with the attention mechanism reduces the coupling between the classification and regression tasks to a certain extent, thus reducing the probability of false and missed detection of the object detection network on various types of targets. Next, we will conduct a series of ablation experiments to demonstrate the effectiveness of the designed anchor-based and anchor-free double branches and the decoupled head structure.

### 4.3 Ablation experiments

In order to verify the effectiveness of the proposed method, a series of ablation experiments are conducted on the basis of the proposed method, keeping both the hardware device and the algorithmic framework unchanged. The ablation experiments mainly explore the role of anchor-based and anchor-free dual branch structure, decoupled detection head with coordinate attention moudle, in our network. Table 2 gives the statistical results of the map metrics and recall metrics for the various methods when the ablation experiments were performed. Rows 3 to 4 in Table 2 indicate the replacement of our proposed double branch structure with the anchor-based structure, the removal of the coordinate attention module from the decoupling header, and the replacement of our proposed decoupling header with the detection header of YOLOV5, respectively.

**Table 2:** The result of the ablation experiments.

method	map_0.5	map_0.5:0.95	recall
Ours	0.78897	0.49976	0.79689
Replace double branch with anchor-based	0.78087	0.49076	0.78659
Without Coordinate Attention Module	0.78657	0.49506	0.79079
Replace decoupling head with YOLOV5 head	0.78207	0.49056	0.78319

From the results in Table 2, we can see that the map_0.5 metric decreases by 0.81 percentage points, the map_0.5:0.95 metric decreases by 0.9 percentage points, and the recall metric decreases by 1.03 percentage points when replacing our designed double branch with anchor-based. This is mainly because the single anchor-based structure lacks anchor-free branches compared to the double branch structure we designed. The single anchor-based branch is only able to detect some regular scale targets, while it often causes false detections and missed detections for extremely small targets and extremely large targets, which affects the overall detection accuracy. In the decoupling header we designed, the map_0.5 metric drops by 0.24 percentage points, the map_0.5:0.95 metric drops by 0.41 percentage points, and the recall metric drops by 0.61 percentage points when the coordinate attention module is removed. This is mainly because Coordinate Attention Module aggregates input features along vertical and horizontal directions into two separate direction-aware feature maps by embedding location information into channel attention. In this way it is possible to more accurately locate and identify defective target areas and improve detection accuracy. When replacing the decoupled head of our design with the standard YOLOV5 head, the map_0.5 metric decreases by 0.69 percentage points, the map_0.5:0.95 metric decreases by 0.92 percentage points, and the recall metric decreases by 1.37 percentage points. This is mainly because our designed decoupling head decouples the regression task and classification task of object detection to a certain extent compared to some coupled structure detection heads including YOLOV5. This decoupled structure reduces the conflict between the two tasks and thus improves the accuracy of object detection. The results of the above ablation experiments prove that the proposed methods are all effective and can be better applied to the multi-scale, defect-irregular scenarios of electronic panel surface defects.

## 5. Conclusion

In this paper, we rethink the mechanistic mechanism of object detection based on the multi-scale problem in electronic panel surface defects, and design the double branch structure. This two-branch structure generates more accurate prediction box and optimizes the feature selection mechanism, provides good detection on multi-scale target defects, and improves the redundancy capability of the network when detecting different targets. Meanwhile, the designed decoupled detection head with coordinate attention module decouples the classification task and regression task of object detection, and this improvement is applicable to all types of defect targets. The final results of both the comparison and ablation experiments prove that our proposed method is effective and has a significant improvement in detection compared to other mainstream networks on the electronic panel surface defect dataset. We hope this paper can bring some inspiration to the defect detection research work in industry or academia. We also see that our network has some room for improvement when facing the problem of sample imbalance in the dataset. Next, we will further improve our network for this type of target and will do further research in unsupervised detection, expecting to further improve the efficiency of defect detection and make some contribution to the industry.

## References

[1] Girshick, R., Donahue, J., Darrell, T., & Malik, J. (2014). Rich feature hierarchies for accurate object detection and semantic segmentation. In *Proceedings of the IEEE conference on computer vision and pattern recognition* (pp. 580–587).

[2] Girshick, R. (2015). Fast r-cnn. In *Proceedings of the IEEE international conference on computer vision* (pp. 1440–1448).

[3] Ren S., He K., Girshick R., & Sun J. (2015). Faster r-cnn: Towards real-time object detection with region proposal networks. *Advances in neural information processing systems*, 28. 10.1109/TPAMI.2016.2577031 27295650

[4] He, K., Gkioxari, G., Dollár, P., & Girshick, R. (2017). Mask r-cnn. In *Proceedings of the IEEE international conference on computer vision* (pp. 2961–2969).

[5] Redmon, J., Divvala, S., Girshick, R., & Farhadi, A. (2016). You only look once: Unified, real-time object detection. In *Proceedings of the IEEE conference on computer vision and pattern recognition* (pp. 779–788).

[6] Redmon, J., & Farhadi, A. (2017). YOLO9000: better, faster, stronger. In *Proceedings of the IEEE conference on computer vision and pattern recognition* (pp. 7263–7271).

[7] Redmon, J., & Farhadi, A. (2018). Yolov3: An incremental improvement. *arXiv preprint arXiv*:*1804*.*02767*.

[8] Bochkovskiy, A., Wang, C. Y., & Liao, H. Y. M. (2020). Yolov4: Optimal speed and accuracy of object detection. *arXiv preprint arXiv*:*2004*.*10934*.

[9] Liu, W., Anguelov, D., Erhan, D., Szegedy, C., Reed, S., Fu, C. Y., et al. (2016, October). Ssd: Single shot multibox detector. In *European conference on computer vision* (pp. 21–37). Springer, Cham.

[10] Lin, T. Y., Maire, M., Belongie, S., Hays, J., Perona, P., Ramanan, D., et al. (2014, September). Microsoft coco: Common objects in context. In *European conference on computer vision* (pp. 740–755). Springer, Cham.

[11] Everingham M., Van Gool L., Williams C. K., Winn J., & Zisserman A. (2010). The pascal visual object classes (voc) challenge. *International journal of computer vision*, 88(2), 303–338.

[12] Dai, X., Chen, Y., Xiao, B., Chen, D., Liu, M., Yuan, L., et al. (2021). Dynamic head: Unifying object detection heads with attentions. In *Proceedings of the IEEE/CVF Conference on Computer Vision and Pattern Recognition* (pp. 7373–7382).

[13] Zhang, H., Li, F., Liu, S., Zhang, L., Su, H., Zhu, J., et al. (2022). DINO: DETR with Improved DeNoising Anchor Boxes for End-to-End Object Detection. *arXiv preprint arXiv*:*2203*.*03605*.

[14] Xie F., Shi M., Shi Z., Yin J., & Zhao D. (2017). Multilevel cloud detection in remote sensing images based on deep learning. *IEEE Journal of Selected Topics in Applied Earth Observations and Remote Sensing*, 10(8), 3631–3640.

[15] Zhu, X., Lyu, S., Wang, X., & Zhao, Q. (2021). TPH-YOLOv5: Improved YOLOv5 Based on Transformer Prediction Head for Object Detection on Drone-captured Scenarios. In *Proceedings of the IEEE/CVF International Conference on Computer Vision* (pp. 2778–2788).

[16] Jing J., Wang Z., Rätsch M., & Zhang H. (2022). Mobile-Unet: An efficient convolutional neural network for fabric defect detection. *Textile Research Journal*, 92(1–2), 30–42.

[17] Howard, A. G., Zhu, M., Chen, B., Kalenichenko, D., Wang, W., Weyand, T., et al. (2021). MobileNets: efficient convolutional neural networks for mobile vision applications, arXiv. *arXiv preprint arXiv*:*1704*.*04861*.

[18] Ronneberger, O., Fischer, P., & Brox, T. (2021). U-Net: convolutional networks for biomedical image segmentation. ArXiv150504597 Cs. Published online May 18, 2015.

[19] He Y, Song K, Meng Q, et al. An end-to-end steel surface defect detection approach via fusing multiple hierarchical features[J]. IEEE Transactions on Instrumentation and Measurement, 2019, 69(4): 1493–1504.

[20] Wang T, Chen Y, Qiao M, et al. A fast and robust convolutional neural network-based defect detection model in product quality control[J]. The International Journal of Advanced Manufacturing Technology, 2018, 94(9): 3465–3471.

[21] Qiu L, Wu X, Yu Z. A high-efficiency fully convolutional networks for pixel-wise surface defect detection [J]. IEEE Access, 2019, 7: 15884–15893.

[22] Zhao Z, Li B, Dong R, et al. A surface defect detection method based on positive samples[C]//Pacific Rim International Conference on Artificial Intelligence. Springer, Cham, 2018: 473–481.

[23] Chen Y, Ding Y, Zhao F, et al. Surface Defect Detection Methods for Industrial Products: A Review[J]. Applied Sciences, 2021, 11(16): 7657.

[24] Mei S, Yang H, Yin Z. An unsupervised-learning-based approach for automated defect inspection on textured surfaces [J]. IEEE Transactions on Instrumentation and Measurement, 2018, 67(6): 1266–1277.

[25] Di H, Ke X, Peng Z, et al. Surface defect classification of steels with a new semi-supervised learning method[J]. Optics and Lasers in Engineering, 2019, 117: 40–48.

[26] Mujeeb A, Dai W, Erdt M, et al. Unsupervised surface defect detection using deep autoencoders and data augmentation[C]//2018 International Conference on Cyberworlds (CW). IEEE, 2018: 391–398.

[27] Hu G, Huang J, Wang Q, et al. Unsupervised fabric defect detection based on a deep convolutional generative adversarial network[J]. Textile Research Journal, 2020, 90(3–4): 247–270.

[28] Gao Y, Gao L, Li X, et al. A semi-supervised convolutional neural network-based method for steel surface defect recognition[J]. Robotics and Computer-Integrated Manufacturing, 2020, 61: 101825.

[29] Hajizadeh S, Núnez A, Tax D M J. Semi-supervised rail defect detection from imbalanced image data[J]. IFAC-PapersOnLine, 2016, 49(3): 78–83.

[30] Lin, T. Y., Goyal, P., Girshick, R., He, K., & Dollár, P. (2017). Focal loss for dense object detection. In *Proceedings of the IEEE international conference on computer vision* (pp. 2980–2988).

[31] Lin, T. Y., Dollár, P., Girshick, R., He, K., Hariharan, B., & Belongie, S. (2017). Feature pyramid networks for object detection. In *Proceedings of the IEEE conference on computer vision and pattern recognition* (pp. 2117–2125).

[32] Huang, L., Yang, Y., Deng, Y., & Yu, Y. (2015). Densebox: Unifying landmark localization with end to end object detection. *arXiv preprint arXiv*:*1509*.*04874*.

[33] Law, H., & Deng, J. (2018). Cornernet: Detecting objects as paired keypoints. In Proceedings *of the European conference on computer vision (ECCV)* (pp. 734–750).

[34] Zhou, X., Zhuo, J., & Krahenbuhl, P. (2019). Bottom-up object detection by grouping extreme and center points. In *Proceedings of the IEEE/CVF conference on computer vision and pattern recognition* (pp. 850–859).

[35] Tian, Z., Shen, C., Chen, H., & He, T. (2019). Fcos: Fully convolutional one-stage object detection. In *Proceedings of the IEEE/CVF international conference on computer vision* (pp. 9627–9636).

[36] Duan, K., Bai, S., Xie, L., Qi, H., Huang, Q., & Tian, Q. (2019). Centernet: Keypoint triplets for object detection. In *Proceedings of the IEEE/CVF international conference on computer vision* (pp. 6569–6578).

[37] Zhou, X., Wang, D., & Krähenbühl, P. (2019). Objects as points. *arXiv preprint arXiv*:*1904*.*07850*.

[38] Liu, W., Hasan, I., & Liao, S. Center and Scale Prediction: A Box-free Approach for Pedestrian and Face Detection. arXiv 2019. *arXiv preprint arXiv*:*1904*.*02948*.

[39] Song, G., Liu, Y., & Wang, X. (2020). Revisiting the sibling head in object detector. In *Proceedings of the IEEE/CVF Conference on Computer Vision and Pattern Recognition* (pp. 11563–11572).

[40] Guo, J., Han, K., Wang, Y., Wu, H., Chen, X., Xu, C., et al. (2021). Distilling object detectors via decoupled features. In *Proceedings of the IEEE/CVF Conference on Computer Vision and Pattern Recognition* (pp. 2154–2164).

[41] Wu, Y., Chen, Y., Yuan, L., Liu, Z., Wang, L., Li, H., et al. (2020). Rethinking classification and localization for object detection. In *Proceedings of the IEEE/CVF conference on computer vision and pattern recognition* (pp. 10186–10195).

[42] He J., Erfani S., Ma X., Bailey J., Chi Y., & Hua X. S. (2021). Alpha-IoU: A Family of Power Intersection over Union Losses for Bounding Box Regression. *Advances in Neural Information Processing Systems*, 34.

